# The Effects of 1,4-Naphthoquinone (NQ) and Naphthazarin (5,8-Dihydroxy-1,4-naphthoquinone, DHNQ) Individually and in Combination on Growth and Oxidative Stress in Maize (*Zea mays* L.) Seedlings

**DOI:** 10.3390/plants12040900

**Published:** 2023-02-16

**Authors:** Waldemar Karcz, Zbigniew Burdach, Małgorzata Rudnicka

**Affiliations:** Institute of Biology, Biotechnology and Environmental Protection, Faculty of Natural Sciences, University of Silesia, Jagiellońska 28, PL-40032 Katowice, Poland

**Keywords:** 1,4-naphthoquinone, naphthazarin, growth, oxidative stress, maize

## Abstract

This study investigated the effects of 1,4-naphthoquinone (NQ) and naphthazarin (5,8-dihydroxy-1,4-naphthoquinone, DHNQ) individually and in combination, applied at low concentrations (0.1, 1, and 10 nM), on growth, hydrogen peroxide (H_2_O_2_) production, catalase activity, and lipid peroxidation in maize seedlings. It was found that NQ at 0.1 and 1 nM and DHNQ at 0.1 nM significantly stimulated the fresh weight of the aboveground parts of the seedlings (APS), while the fresh weight of the underground parts of the seedlings (UPS) was enhanced only at 0.1 nM NQ. Interestingly, DHNQ at higher concentrations (1 and 10 nM) significantly diminished the fresh weight of the APS and UPS. When NQ and DHNQ were applied together, an increase in the fresh weight of the APS at all of the concentrations studied was observed. It was also found that NQ and DHNQ individually and in combination, at all concentrations studied, decreased the H_2_O_2_ production in the aboveground and underground parts of maize seedlings. The presence of the DHNQ at higher concentrations (1 and 10 nM) triggered an increase in the catalase (CAT) activity of the UPS and APS compared to the control. However, NQ added at 1 nM decreased the CAT activity of both the UPS and APS, while 10 nM increased the CAT activity of UPS. NQ and DHNQ applied together at 0.1 and 10 nM almost completely inhibited catalase activity in the UPS and APS. The data that were obtained for lipid peroxidation, measured as the malondialdehyde (MDA) concentration, indicated that NQ and DHNQ at all concentrations studied decreased the MDA content of the UPS, while both naphthoquinones increased it in APS. The data presented here are discussed taking into account the mechanisms via which naphthoquinones interact with biological systems.

## 1. Introduction

Naphthoquinones are the products of bacterial and fungal metabolism, as well as secondary metabolism in higher plants, where they are produced and used as natural defense chemicals [1,2,3,4]. Naturally occurring naphthoquinones, such as juglone (5-hydroxy-1,4-naphthoquinone), lawsone (2-hydroxy-1,4-naphthoquinone), plumbagin (2-methyl-5-hydroxy-1,4-naphthoquinone), naphthazarin (5,8-dihydroxy-1,4-naphthoquinone), and others, have been studied for a long time due to their importance in medicinal and biological research [5,6,7,8,9], and recently in the energy and chemical industries [10,11,12]. Interestingly, in the last two cases, naphthoquinone can be used as an organic component of the positive electrode in an ecological battery or as a natural corrosion inhibitor. Naphthoquinones are referred to as allelochemicals, which, in agreement with the definition proposed by Dayan et al. [7], indicate phytotoxins that may negatively influence vegetation in the vicinity of the producing plant.

**Figure 1:**
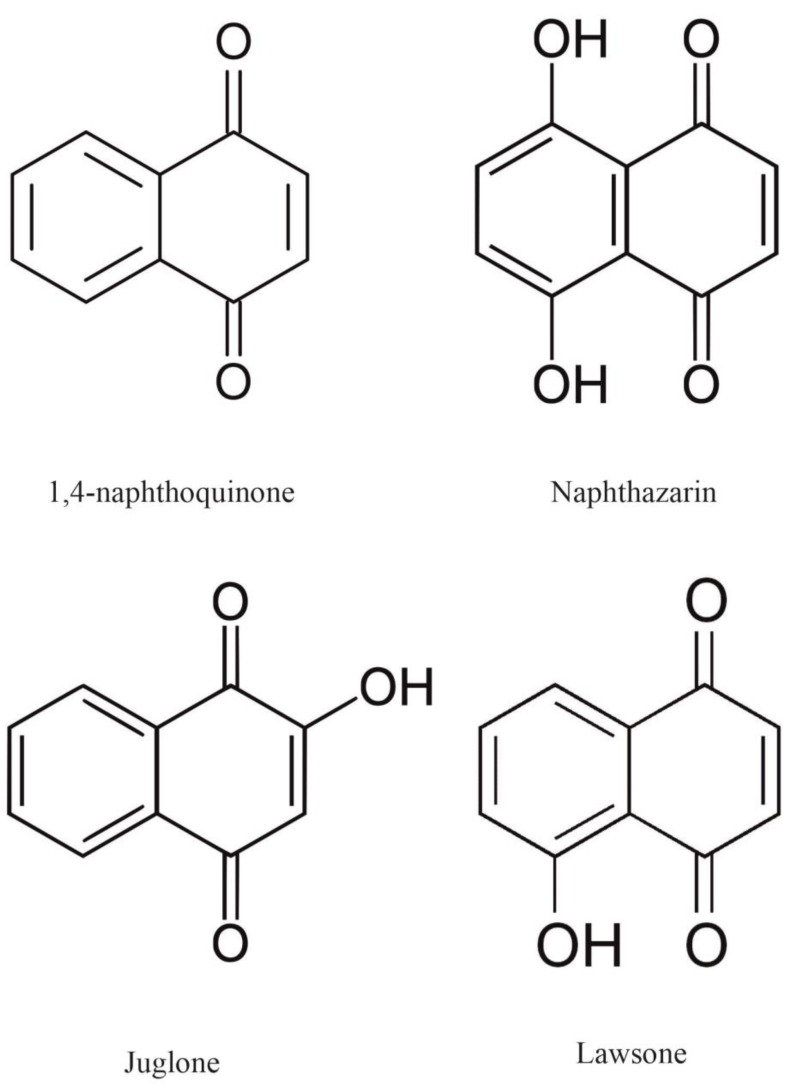
The chemical structures of 1,4-naphthoquinone (NQ) and its derivative 5,8-dihydroxy-1,4-naphthoquinone (naphthazarin, DHNQ). 1,4-NQ is structurally related to naphthalene and consists of two rings that are fused together, i.e., benzene and quinone, in which the carbonyl groups are in the para position. The effects of juglone (5-hydroxy-1,4-naphthoquinone) and lawsone (2-hydroxy-1,4-naphthoquinone) on the growth of plant cells were studied in our earlier experiments [15,16,17,18]; hence, their chemical structures are also shown.

Naphthazarin is one of the natural 1,4-naphthoquinone substances derived from the tissues of several members of the *Boraginaceae, Droseraceae*, and *Nepenthaceae* families [13,14] (Figure 1).

Naphthazarin and its derivatives have a wide variety of pharmacological activities, including anticancer, anti-inflammatory, antibacterial, and antifungal effects [9,19,20,21,22]. Naphthoquinones and other plant secondary metabolites can potentially be used as biopesticides and bioherbicides due to the multiplicity of their modes of action (MOAs) compared to traditional plant protection products. There are many examples of strong natural phytotoxins with MOAs that are not utilized by commercial herbicides (reviewed in [23,24,25,26]).

There are two general action mechanisms of naphthoquinones; one is the covalent modification of biological molecules at their nucleophilic sites, such as the thiols in proteins and glutathione (GSH), in which quinones act as electrophiles, while the other mechanism consists of redox cycling, in which reactive oxygen species (ROS) are generated [4,27,28,29]. However, Rudnicka et al. [17,18] recently suggested that naphthazarin may directly inhibit the PM H^+^-ATPase activity via an arylation process where it reacts with the thiol groups of the protein or indirectly by producing ROS via redox cycling. In addition, these authors also showed that naphthazarin (5,8-dihydroxy-1,4-naphthoquinone) changed the auxin-induced reorientation of the cortical microtubules from perpendicular to oblique with respect to the long cell axis, which, according to the authors, suggests that this effect may be related to the nucleophilic interaction of naphthazarin with the proteins that are associated with the cortical microtubule reorientation processes.

Bioassays that use plant growth and other physiological processes are often the primary tools for determining the biological activity of natural and synthetic compounds. The main goal of the present study was to determine the effects of 1,4-naphthoquinone (NQ) and naphthazarin (5,8-dihydroxy-1,4-naphthoquinone, DHNQ) individually and in combination, both at low concentrations (0.1, 1, and 10 nM), on the growth of maize seedlings and shed light on the mechanism of this phenomenon. This goal was realized by (1) studying the effect of both naphthoquinones and their combination on the length of maize seedling organs (main root, mesocotyl, and first leaf) and fresh weight of the above- and underground parts of the seedlings, (2) determining the redox cycling properties of NQ and DHNQ individually and in combination (estimated by H_2_O_2_ production and catalase activity) in maize seedlings, and (3) examining the effects of NQ, DHNQ, and NQ plus DHNQ on the malondialdehyde (MDA) content of maize seedlings. This experimental design can provide new data on the effects of naphthoquinones on the growth of plant seedlings.

## 2. Results

### 2.1. The Effects of NQ and DHNQ Individually and in Combination on the Growth and Fresh Weight of Maize Seedlings

**Figure 2:**
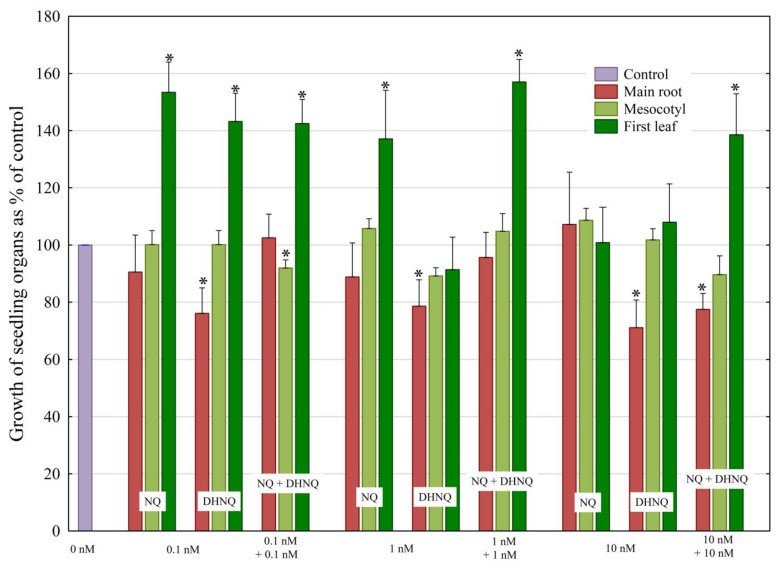
The effects of naphthoquinones (NQ and DHNQ) individually and in combination on the lengths of maize (*Zea mays* L.) seedling organs (main root, mesocotyl, and first leaf), shown as a percentage of the control (100%, seedlings not treated with naphthoquinones). The data presented are the means of three independent experiments (nine seedlings in each). Bars indicate means ± SEs. Mean length in the control: main root, 236.6 mm; mesocotyl, 71.6 mm; first leaf, 214.1 mm. The normal distribution was evaluated using the Shapiro–Wilk test (*p* > 0.05). The statistical differences between the control and other variants were analyzed using a paired *t*-test (*p* < 0.05). Asterisks (*) indicate a significant difference between the control and other variants.

The concentrations of the naphthoquinones used here (0.1, 1, and 10 nM) were selected taking into account our previous papers [16,17,18] in which the effects of 1,4-naphthoquinone, lawsone, and naphthazarin on the elongation growth of maize coleoptile cells were studied. Data in Figure 2 indicate that the growth of the seedlings treated with the naphthoquinones individually and in combination depended on the amount and type of the naphthoquinones used.

The treatment of maize seedlings with 1,4-naphthoquinone (NQ) at concentrations of 0.1, 1, and 10 nM did not significantly change the growth of the main roots and mesocotyls, while, at 0.1 nM and 1 nM, NQ stimulated the growth of the first leaves by about 40–50% (Figure 2). In the case of naphthazarin (DHNQ), the growth of the roots was inhibited by 20–30% at all concentrations studied. DHNQ at 0.1 nM, similarly to NQ, stimulated the growth of the first leaves, but did not significantly change it at higher concentrations (1 and 10 nM). When NQ and DHNQ were added together at the same concentrations (1 nM), the effect of DHNQ on the growth of the seedling roots and leaves was inhibited, while the growth of leaves was significantly stimulated (by ca. 60%) compared to the control. A similar effect was observed when both naphthoquinones were added at 10 nM, except that the presence of NQ in the mixture did not inhibit the toxic effect of DHNQ on root growth.

**Figure 3:**
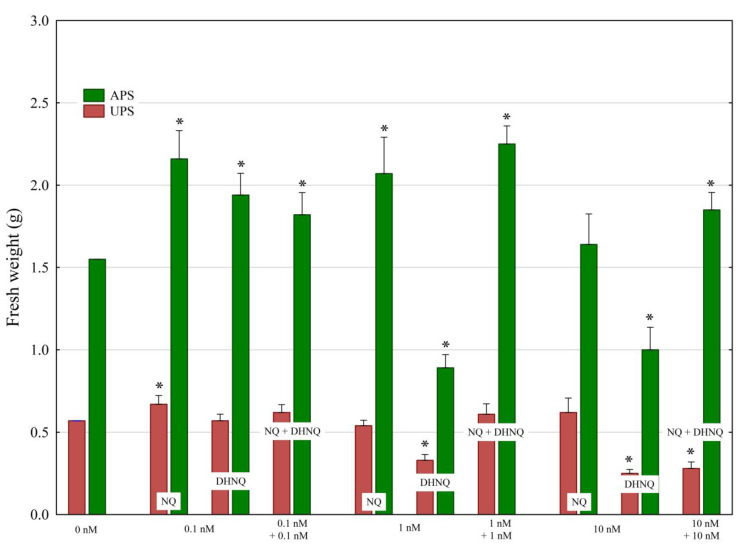
The effects of naphthoquinones (NQ and DHNQ) individually and in combination on the fresh weight of the aboveground parts of the seedlings (APS) and underground parts of the maize seedlings (UPS) compared to the control (seedlings not treated with naphthoquinones). The data presented are the means of three independent experiments (nine seedlings in each). Bars indicate means ± SEs. Mean fresh weight in the control: UPS, 0.57 g; APS, 1.55 g. Asterisks (*) indicate a significant difference between the control and other variants.

In order to more quantitatively verify the results obtained from the measurements of the elongation growth of the seedling organs, the fresh weight of the aboveground parts of the seedlings (APS) and underground parts of the seedlings (UPS) was also determined (Figure 3). In subsequent studies on oxidative stress, only the fresh weight is taken into account.

The treatment of maize seedlings with NQ at 0.1 and 1 nM stimulated a ca. 40% increase in the fresh weight of the aboveground parts of the seedlings (APS) compared to the control, while 10 nM NQ did not significantly change it. In the case of DHNQ, the fresh weight of the APS was stimulated by 20% only at 0.1 nM; however, at higher concentrations, 1 and 10 nM, DHNQ did not change the fresh weight of the APS and inhibited it (by ca. 40%), respectively, compared to the control. When NQ and DHNQ were added together at 1 nM, a stimulatory effect of both naphthoquinones on the fresh weight of the (APS) was observed; however, when NQ and DHNQ were added together at 10 nM, the toxic effect of DHNQ on the fresh weight of the APS was inhibited. In the case of the fresh weight of the underground part of the seedlings (UPS), an increase was observed only in the presence of 0.1 nM NQ, while, at higher concentrations, NQ did not change this parameter. DHNQ significantly lowered the fresh weight of the UPS at higher concentrations (1 and 10 nM). However, when NQ and DHNQ were added together at 1 and 10 nM, the toxic effect of DHNQ, in contrast to the fresh weight of the APS, was inhibited only at 1 nM DHNQ.

### 2.2. The Effects of NQ and DHNQ Individually and in Combination on the Production of H_2_O_2_ and Catalase Activity in the Aboveground and Underground Parts of Maize Seedlings

**Figure 4:**
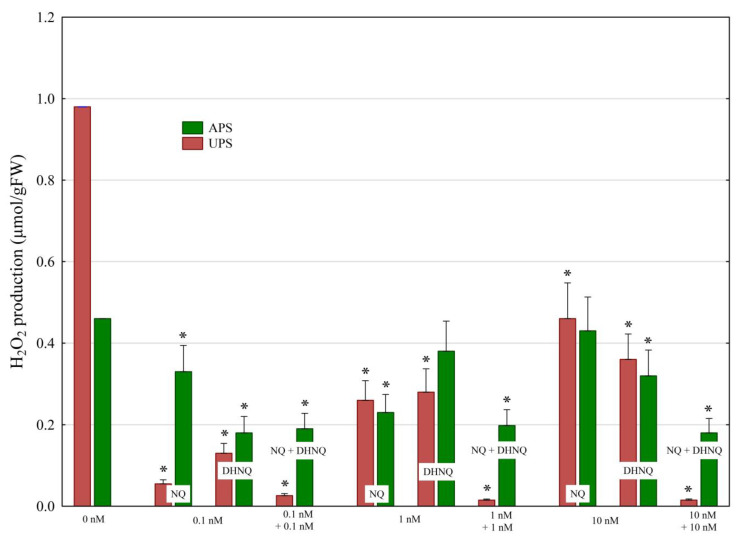
The effects of naphthoquinones (NQ and DHNQ) individually and in combination on the H_2_O_2_ production (µmol/g FW) in maize seedlings. Mean values of H_2_O_2_ production (µmol/g FW) in the control: UPS, 0.98 μmol/g FW; APS, 0.46 μmol/gFW. Asterisks (*) indicate a significant difference between the control and other variants.

The addition of NQ and DHNQ individually and in combination to the control medium, at all concentrations studied, resulted in a decrease in hydrogen peroxide production (H_2_O_2_) to lower values compared to the control medium (Figure 4).

In all experimental variants, the production of H_2_O_2_ in the aboveground parts of the seedlings (APS) was greater than that in the underground parts of the seedlings (UPS). For example, when NQ or DHNQ was added at concentrations higher than 0.1 nM, the production of H_2_O_2_ in the APS was at least twofold greater than in the UPS. DHNQ added at concentrations higher than 0.1 nM enhanced H_2_O_2_ production, although it was still lower than in the control. The same was observed for NQ added at 10 nM. Interestingly, NQ and DHNQ added together at all of the concentrations studied almost completely inhibited the production of H_2_O_2_ in the UPS, while, for APS, the production of H_2_O_2_ was decreased to values lower by almost 60% compared to the control.

**Figure 5:**
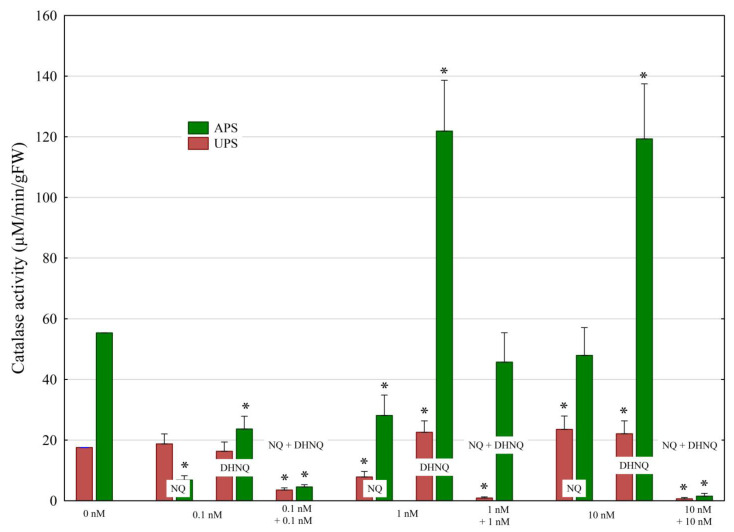
The effects of naphthoquinones (NQ and DHNQ) individually and in combination on the catalase activity (µmol H_2_O_2_/min/g FW) in maize seedlings. Mean values of the catalase activity in the control: UPS, 17.56 μmol H_2_O_2_/min/gFW; APS, 55.38 μmol H_2_O_2_/min/gFW. Asterisks (*) indicate a significant difference between the control and other variants.

Catalase plays a key role in the elimination of oxygen radicals by eliminating H_2_O_2_. The presence of NQ or DHNQ in the control medium at the lowest concentration (0.1 nM) did not change the catalase (CAT) activity in the UPS, whereas it significantly reduced it in the APS compared to the control (Figure 5).

NQ and DHNQ applied together at 0.1 nM almost completely inhibited catalase activity (CAT) in the UPS and APS compared to the control. The presence of DHNQ at 1 and 10 nM triggered an increase in the catalase activity of the UPS and APS by about 20% and 100%, respectively, compared to the control. However, NQ added at the same concentrations as DHNQ at 1 nM decreased the CAT activity of both the UPS and the APS by ca. 50%, while 10 nM increased the CAT activity of the UPS. NQ and DHNQ added in combination inhibited the CAT activity at almost all concentrations tested, excluding variant NQ + DHNQ at 1 nM for APS (Figure 5).

### 2.3. The Effects of NQ and DHNQ Individually and in Combination on Lipid Peroxidation (MDA) in Maize Seedlings

**Figure 6:**
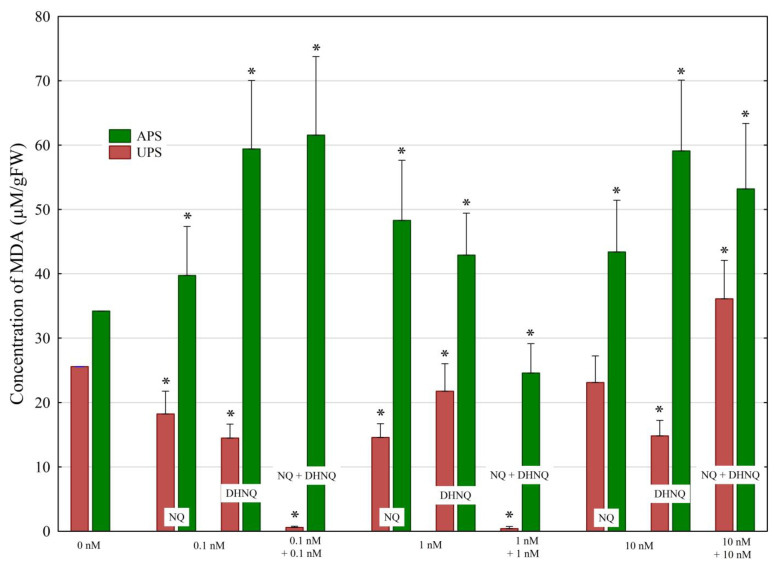
The effects of naphthoquinones (NQ and DHNQ) individually and in combination on the MDA content (µmol/g FW) in maize seedlings. Mean values of the MDA content (µmol/g FW) in the control seedlings: UPS, 25.6 µmol/g FW; APS, 34.2 µmol/g FW. Asterisks (*) indicate a significant difference between the control and other variants.

Lipid peroxidation in the UPS and APS treated with NQ and DHNQ individually and in combination, which was measured as the malondialdehyde (MDA) concentration, is presented in Figure 6.

The data obtained indicate that NQ and DHNQ at all concentrations studied decreased the MDA content of the UPS, while they increased the MDA content of the APS compared to the control (seedlings not treated with NQ or DHNQ). Interestingly, DHNQ at 0.1 and 10 nM increased the MDA content of APS much more than NQ applied at the same concentrations. When both naphthoquinones were added together at 0.1 and 1 nM, their combined effect resulted in both a decrease in MDA content of the UPS to values close to 0% and a decrease in MDA content of the APS compared to the control. The combined effect of both naphthoquinones applied at 10 nM resulted in stimulation of the UPS and APS by ca. 40% and 60%, respectively, compared to the control.

### 2.4. A Comparison of the Effects of 1,4-Naphthoquinone (NQ) and Naphthazarin (5,8-Dihydroxy-1,4-naphthoquinone, DHNQ) Individually and in Combination on the Fresh Weight, H_2_O_2_ Production, Catalase Activity, and Lipid Peroxidation in Maize Seedlings

Table 1 compares the parameters determined for maize seedlings treated with naphthoquinones and their combinations.

Considering the data presented in Table 1, it should be noted that NQ at lower concentrations (0.1 and 1 nM) caused an increase in the fresh weight of the APS, accompanied by a decrease in the production of H_2_O_2_ and catalase activity, while NQ at 10 nM only significantly changed the lipid peroxidation. NQ at all concentrations studied caused an increase in lipid peroxidation (measured as MDA concentrations). In contrast to NQ, DHNQ at higher concentrations (1 and 10 nM) decreased the fresh weight of the APS, accompanied by a decrease in the production of H_2_O_2_ and an increase in catalase activity. As in the case of NQ, DHNQ enhanced lipid peroxidation. When NQ and DHNQ were applied together, an increase in the fresh weight of the APS at all concentrations studied was observed. As in the case of NQ, the increase in fresh weight was accompanied by a marked decrease in H_2_O_2_ production and catalase activity. The combination of both naphthoquinones at 0.1 and 10 nM significantly stimulated the MDA content of the APS.

Comparing the abovementioned data obtained for APS with those for UPS, it should be stated that NQ at 0.1 nM increased the fresh weight of the UPS, while, at higher concentrations, no change was observed. In turn, DHNQ at 1 and 10 nM, similar to the case of the APS, diminished the fresh weight of the UPS. NQ and DHNQ applied together at lower concentrations slightly stimulated the fresh weight of the UPS. As in the case of the APS, the production of H_2_O_2_ in the UPS was reduced, especially under the combined action of NQ and DHNQ, where it was practically reduced to zero. Moreover, as in the case of the APS, catalase activity in the UPS was stimulated at higher concentrations of DHNQ (1 and 10 nM) and inhibited, especially in the case of the combined action of NQ and DHNQ. In contrast to APS, the MDA concentration in the UPS was decreased at all concentrations of NQ and DHNQ individually and in combination, excluding the variant of NQ and DHNQ added together at 10 nM.

## 3. Discussion

In recent years, a great deal of attention has been paid to naturally occurring naphthoquinones as compounds with a high level of biological activity. The toxicity of naphthoquinones is mainly due to two major types of reaction. First, as redox cyclers, they induce oxidative stress by generating reactive oxygen species (ROS); second, as electrophiles, they react with nucleophiles, such as thiols or glutathione [4,29]. It is rather well established that most plant allelochemicals produce inhibitory effects at higher doses, but they can also function as biostimulants at lower ones. In the latter case, this phenomenon is known as hormesis (reviewed in [30]).

Rudnicka et al. [16,17,18] recently showed that 1,4-naphthoquinone (NQ), lawsone (NQ-2-OH), and naphthazarin (5,8-dihydroxy-1,4-naphthoquinone, DHNQ), applied at low concentrations (1 and 10 nM), had a toxic effect on maize coleoptile segments by inhibiting endogenous and IAA-induced growth, as well as proton extrusion. The data presented in these papers clearly demonstrated that, at lower concentrations, the abovementioned naphthoquinones enhanced the H_2_O_2_ production in maize coleoptile cells, which closely contributed with an increase in catalase activity (CAT) and lipid peroxidation. Interestingly, when 1,4-naphthoquinone (NQ) and naphthazarin (DHNQ) were applied together at 1 and 10 nM, their inhibitory effect on the auxin-induced growth and proton extrusion of the maize coleoptile cells was stronger compared to the individual action of either of the naphthoquinones. In the presence of both naphthoquinones at 1 nM, the oxidative stress was significantly higher. It should be added here that the segments of etiolated maize coleoptiles (cut 3–5 mm below the tip of the coleoptiles) represent a classical model system for studies on the mechanism of auxin action on plant cell growth. In this system, the number of cells is constant, and an organ grows only through elongation. Interestingly, most of the important evidence on the molecular mechanisms of auxin action was obtained in experiments performed with segments of maize coleoptiles (reviewed in [31]).

The main goal of the experiments described here was to shed light on the effects of 1,4-naphthoquinone (NQ) and its derivative naphthazarin (5,8-dihydroxy-1,4-naphthoquinone, DHNQ) individually and in combination, applied at low concentrations (0.1, 1, and 10 nM), on the growth and oxidative stress in the intact maize seedlings. Understanding these effects is important due to the allelochemical properties of naphthoquinones and their effect on plant growth and development.

Taking into account the data presented above for the fresh weight, it can be suggested that NQ at 0.1 and 1 nM and DHNQ at 0.1 nM significantly stimulated the fresh weight of the APS, while, in the UPS, it was changed only slightly. Interestingly, DHNQ at higher concentrations (1 and 10 nM) significantly diminished the fresh weight of the APS and UPS. The observed differences between APS and UPS probably resulted, among other reasons, from the fact that, in the case of the UPS, the naphthoquinones directly interacted with the root system. This means that NQ and DHNQ could change root growth by affecting two processes: cell division and cell expansion. However, in order to at least partially explain the mechanisms via which naphthoquinones induced changes in the fresh weight of the seedlings, we also took into account the redox cycling properties of both naphthoquinones, determined as the H_2_O_2_ production, catalase activity, and lipid peroxidation in maize seedlings.

The data presented in Figure 4 and Table 1 demonstrate that NQ and DHNQ individually and in combination, at all concentrations studied, decreased the H_2_O_2_ production in the aboveground and underground parts of maize seedlings compared to the control (Figure 4). The high H_2_O_2_ in control plants is due the fact that H_2_O_2_ is produced predominantly in plants during photosynthesis and photorespiration. Interestingly, NQ and DHNQ added together almost completely inhibited the production of H_2_O_2_ in the UPS, while, in the APS, it was decreased to values lower by almost 60% compared to the control (Figure 4 and Table 1). This observation suggests that both naphthoquinones, individually and in combination, decreased and completely inhibited the production of H_2_O_2_ in maize seedlings. This finding may suggest the antioxidant properties of NQ and DHNQ individually and in combination. Such properties were previously demonstrated for juglone (5-hydroxy-1,4-naphthoquinone) by others [32,33,34] (for a review, see also [3]).

Catalase (CAT) plays a key role in maintaining the balance between H_2_O_2_ production and removal, which is essential for the survival of plants [35,36]. The presence of DHNQ at higher concentrations (1 and 10 nM) triggered an increase in the catalase activity of the UPS and APS by about 30% and 120%, respectively, compared to the control. At the same concentrations of DHNQ, the H_2_O_2_ production was reduced by about 70% and 25%, respectively, while, at both concentrations of DHNQ. the fresh weight of the UPS and APS was decreased by about 40%. However, NQ added at the same concentrations as DHNQ at 1 nM decreased the CAT activity of both the UPS and the APS by ca. 60%, while 10 nM increased the CAT activity of UPS. NQ and DHNQ applied together at 0.1 and 10 nM almost completely inhibited catalase activity in the UPS and APS as compared to the control (Figure 5 and Table 1). Due to the lack of relevant data in the literature, it is difficult to compare our results obtained for catalase activity with those of other authors.

Reactive oxygen species (ROS) are products of membrane lipid peroxidation, and MDA content can indicate the degree of lipid peroxidation damage [37]. Under normal growth conditions of plants, ROS are signal molecules involved in various cellular processes [38]. However, like other stress factors, allelochemicals result in the increased production of ROS, which can interfere with different cellular processes, thus causing inhibition of growth [39,40]. The data obtained for lipid peroxidation (measured as the MDA concentration) indicated that NQ and DHNQ, at all concentrations studied, decreased the MDA content of the UPS, while both naphthoquinones increased it in the APS compared to the control. Interestingly, DHNQ at 0.1 and 10 nM increased the MDA content of the APS much more than NQ applied at the same concentrations. When both naphthoquinones were added together at 0.1 and 1 nM, their combined effect resulted in both a decrease in MDA content of the UPS to values close to 0% and a decrease in MDA content of the APS compared to the control. The combined effect of both naphthoquinones applied at 10 nM resulted in UPS and APS stimulation by ca. 40 and 60%, respectively, compared to the control (Figure 6 and Table 1). Our results showed that the MDA content was increased in the APS at almost all concentrations of naphthoquinones studied, while it was decreased in the UPS. The data obtained for lipid peroxidation in maize seedlings indicate that, in almost all variants of the experiments, the content of MDA was negatively correlated with other parameters determined for the aboveground parts of the seedlings. In the case of the underground parts of the seedlings, a positive correlation could only be indicated for catalase activity and lipid peroxidation. Taking into account the values of the individual parameters obtained for the above- and underground parts of the seedlings, it should be stated that the relationships between them are complex and require further research. Considering the results obtained for the above- and underground parts of the seedlings, it must be undoubtedly stated that NQ at 0.1 and 1 nM and DHNQ at 0.1 nM clearly stimulated the growth of APS, while the growth of the underground parts of seedlings slightly changed. There is also no doubt that when NQ and DHNQ were added together, NQ reduced the toxic effects of DHNQ. The obtained results may also provide additional arguments in favor of the hypothesis recently proposed by [4]: “Given the propensity of some 1,4-naphthoquinones to react with free thiols [41], it can be hypothesized that conjugation with GSH and/or free cysteine might also be a strategy that plants use to cope with 1,4 naphthoquinone allelochemicals”.

In conclusion, this study sheds new light on the effects of 1,4-naphthoquinone and naphthazarin alone and in combination on the growth and oxidative stress of maize seedlings, suggesting the use of both naphthoquinones at low concentrations as growth biostimulants.

## 4. Materials and Methods

### 4.1. Plant Material and Growth Conditions

All experiments were conducted on 21 day old maize seedlings grown hydroponically. Briefly, maize (*Zea mays* L. cv. Cosmo 230) caryopses were soaked in tap water for 2 h, sown on wet lignin in plastic containers, and placed in a growth chamber (Type MIR-553, Sanyo Electric Co., Osaka, Japan) at 27 ± 1.0 °C. Four day old seedlings with similar lengths (50–70 mm) were transferred to 2.4 L opaque plastic containers (nine seedlings in each) filled with Hoagland’s solution [42,43]; for the next 3 days, the seedlings were again placed in the growth chamber (for acclimatization). Next, 1 week old seedlings were grown in a greenhouse at a constant temperature of 22 °C and a photoperiod of 14/10 h (light/dark). Hoagland’s medium was replaced at 3 day intervals. Naphthoquinones were added to the Hogland’s medium on day 7 of seedlings grown in the greenhouse (14 day old seedlings), and the seedlings were treated with naphthoquinones within 1 week (Hogland’s medium supplemented with naphthoquinones was replaced at 3 day intervals). The experiments were performed in triplicate.

### 4.2. Growth Measurements

After 21 days of the experiment, the lengths of the maize seedling organs (main root, mesocotyl, and first leaf) were determined with a ruler. The fresh weight of the aboveground and underground parts of the seedlings was then determined using a laboratory balance (AKA220, AXIS, AXIS Sp. z o.o. Gdańsk, Poland).

### 4.3. Hydrogen Peroxide Detection

Hydrogen peroxide (H_2_O_2_) in roots and shoots of maize seedlings was determined according to Velikova et al. [44] and Junglee et al. [45] with slight modifications [17]. In short, after 7 days of treatment of maize seedlings with naphthoquinones, 0.1 g fresh weight of the root or leaf samples were homogenized in 1.5 mL of 0.1% (*w*/*v*) trichloroacetic acid (TCA). The homogenate was centrifuged at 10,000× *g* at 4 °C for 10 min. Then, 0.5 mL of the supernatant was added to 0.5 mL of 0.1 M K-phosphate buffer (pH 7.0) and 1 mL of 1 M KI. The absorbance of the supernatant was measured at 350 nm. The H_2_O_2_ content, expressed in µmol/g fresh weight (FW), was calculated from a standard calibration curve prepared in 0.1% TCA at different H_2_O_2_ concentrations.

### 4.4. Catalase Activity

Catalase activity in 21 day old maize roots and leaves was determined as described by Cavalcanti et al. [46] with slight modifications [17]. Briefly, 0.2 g of a fresh sample was homogenized in 1.5 mL of 0.1 M K-phosphate buffer (pH 7.0). The homogenate was centrifuged at 12,000× g at 25 °C for 20 min. Then, 20 µL of the supernatant was added to 2 mL of 10 mM H_2_O_2_, and the absorbance drop was measured at 240 nm and 30 °C. Enzyme activity was calculated using a molar extinction coefficient of 36 × 10^3^/mM/m and expressed as µmol H_2_O_2_ oxidized/g FW/min.

### 4.5. Estimation of MDA Content

The lipid peroxidation in maize seedlings was determined by estimating the MDA content as a TBARS concentration, described by Hodges et al. [46] with slight modifications [17]. Briefly, 0.5 g samples of maize root or leaf were placed immediately in liquid nitrogen. The plant tissues were then homogenized in 12.5 mL of 80% ethanol. Then, 1 mL of the homogenized sample was added to a tube of 1 mL of (1) −TBA solution consisting of 20% (*w*/*v*) TCA and 0.01% butylated hydroxytoluene, or (2) +TBA solution containing the above plus 0.65% TBA. Samples were then vigorously mixed, heated at 95 °C in a boiling water bath for 20 min, cooled, and centrifuged at 10,000× *g* at 4 °C for 10 min. Absorbance values were read at 440, 532, and 600 nm. MDA equivalents (µmol/g FW) were calculated according to [46].

### 4.6. Statistical Analysis

The data were analyzed using Dell Statistica (data analysis software system), version 13 (Dell, Texas, TX, USA). The normal distribution was evaluated using the Shapiro–Wilk test (*p* > 0.05). The statistical differences between the control and other variants were analyzed using a paired *t*-test (*p* < 0.05).

## Figures and Tables

**Table 1:** The effects of NQ and naphthazarin (DHNQ) individually and in combination on the fresh weight, H_2_O_2_ production, catalase activity, and lipid peroxidation in the aboveground parts of the seedlings (APS) and underground parts of the seedlings (UPS). The data were adopted from Figure 3, Figure 4, Figure 5 and Figure 6. Red color denotes a value above the control.

Tested Parameters	Effect of NQ and DHNQ as Well as Their Combination on Fresh Weight (FW), H_2_O_2_ Production, Catalase Activity and Lipid Peroxidation (MDA) in the Aboveground Parts of the Seedlings (APS) and Underground Parts of the Seedlings (UPS) as % of Control																	
	Naphthoquinones and Their Concentrations (nM)																	
	APS									UPS								
	NQ			DHNQ			NQ + DHNQ			NQ			DHNQ			NQ + DHNQ		
	0.1	1	10	0.1	1	10	0.1	1	10	0.1	1	10	0.1	1	10	0.1	1	10
Fresh Weight (FW)	39.6	33.5	5.8	25.2	−42.6	−35.5	17.4	45.2	19.4	17.5	−5.3	7.7	0	−42.1	−56.2	8.7	7.1	−50.9
H_2_O_2_ Production	−27.7	−51.7	−6.8	−60.8	−17.6	−31.8	−58.3	−56.8	−60.8	−94.4	−73.7	−52.6	−86.8	−71.1	−63.2	−97.3	−98.4	−98.4
Catalase Activity	−87.7	−49.2	−13.4	−57.3	120.1	115.3	−91.8	−17.4	−97.3	6.8	−55.4	34.1	−7.2	28.7	25.8	−79.8	−94.8	−96.3
Lipid Peroxidation (MDA)	16.2	41.2	26.8	73.5	25.5	72.6	80.1	−28.2	55.5	−28.7	−43.1	−9.6	−43.4	−14.9	−42.1	−97.7	−98.4	40.8

Fresh weight: control—100%; UPS, 0.57 g; APS, 1.55 g. H_2_O_2_ production: control—100%; UPS, 0.98 μmol/g FW; APS, 0.46 μmol/gFW. Catalase activity: control—100%; UPS, 17.56 μmol H_2_O_2_/min/gFW; APS, 55.38 μmol H_2_O_2_/min/gFW. MDA content: control—100%; UPS, 25.6 µmol/g FW; APS, 34.2 µmol/g FW.

## Data Availability

Data is contained within the article.
